# Current Trends in Antimicrobial Resistance Patterns in Bacterial Pathogens among Adult and Pediatric Patients in the Intensive Care Unit in a Tertiary Care Hospital in Kolkata, India

**DOI:** 10.3390/antibiotics12030459

**Published:** 2023-02-24

**Authors:** Mandira Chakraborty, Sayani Sardar, Rituparna De, Malabika Biswas, Maria Teresa Mascellino, Maria Claudia Miele, Silpak Biswas, Anita Nandi Mitra

**Affiliations:** 1 Department of Microbiology, Calcutta Medical College and Hospital, Kolkata 700073, India; 2 Department of Microbiology, School of Tropical Medicine, Kolkata 700073, India; 3 Department of Public Health and Infectious Diseases, Sapienza University of Rome, 00185 Rome, Italy

**Keywords:** nosocomial infections, intensive care units, antibiotic resistance, multidrug-resistant bacteria, public health

## Abstract

Nosocomial infections by multidrug-resistant (MDR) bacteria are among the main causes of morbidity and death in patients hospitalized in intensive care units (ICUs) worldwide. Antibiotic resistance has become a major concern for treating the patients with nosocomial infections. The aim of this study was to describe the antibiotic resistance patterns of pathogens causing infections in adult and pediatric patients in the ICUs of a tertiary care hospital in Kolkata, India. A cross-sectional, retrospective study was conducted from January 2022 to October 2022 on a total of 139 adult and 146 pediatric patients. Depending on clinical symptoms of the patients, samples were collected and subjected to antibiotic sensitivity testing. The culture and sensitivity pattern of clinical isolates from blood, urine, sputum, endotracheal tube (ET) aspirate, and central line catheter insertion site swabs were analyzed. A total of 695 and 556 specimens were obtained from adult and pediatric ICU, respectively. Culture positivity rate among adults and pediatric patients were 37% and 40%, respectively. The most commonly isolated organisms were Gram-negative Enterobacterales and non-fermenters. Most of the bacterial isolates showed very high resistance against multiple antibiotics. *Escherichia coli* from adult and pediatricpatients were found to be resistant to second generation cephalosporins (95% and 96%, respectively), beta-lactams (95% and 63%, respectively), fluoroquinolones (95% and 81%, respectively), and cotrimoxazole (85% and 78%, respectively). *Klebsiella* spp. from adult patients were found to be resistant to aminoglycosides (75%), second generation cephalosporins (100%), beta-lactams (94%), fluoroquinolones (92%), carbapenems (88%), and cotrimoxazole (83%). *Proteus* spp., *Acinetobacter baumannii*, and *Pseudomonas* spp. werefound to be resistant to multiple antibiotics. *Enterococcus* spp. from ICUs showed more than 90% resistance against ampicillin and more than 75% resistance against fluoroquinolones. MDR bacterial infections are increasing in both adult and pediatric ICUs, leading to significant therapeutic challenges. A frequent study of antimicrobial resistance patterns is imperative for antibiotic stewardshipin combatting the deadly effect of the MDR bacteria in critically ill patients.

## 1. Introduction

Nosocomial infections are one of the main causes of mortality and morbidity in patients hospitalized in intensive care units (ICUs) [1,2,3]. ICUs are considered as the epicenter of infections because of their vulnerable patient population. The high incidence rates of nosocomial infections in these patients leads to high impact on health care costs [1,4,5]. Some of the major causes of these infections are long duration of hospital stay, empirical use of broad spectrum antibiotics, insertion of invasive devices, and lack of infection control [2,6,7,8]. Previously, some studies have demonstrated various features of essential health impairment, such aschronic lung disease [1,9], immunocompromised state [10], undernourishment [11], etc., as some of the risk factors of nosocomial infections [12]. The majority of the nosocomial infections are caused by the ESKAPE pathogens, as they can“escape” the biocidal action of antibiotics [13,14,15]. The acronym ESKAPE includes six nosocomial pathogens: *Enterococcus faecium, Staphylococcus aureus, Klebsiella pneumoniae, Acinetobacter baumannii, Pseudomonas aeruginosa*, and *Enterobacter* spp. [14,16,17]. According to the report of the European Centre for Disease Prevention and Control, 8% of patients staying in the ICU for >2 days showed at least one ICU-associated nosocomial infections [18]. In developing countries, the burden of nosocomial infections is more significant. For example, in eastern India, a Kolkata based hospital reported the nosocomial infection rate in the ICU to be as high as 11.98% [12]. Recently, Branstetter et al. [19] reported that in United States of America, 2 million pediatric and adult patients develop nosocomial infections each year.

Development and dissemination of antibiotic resistance in intensive care units has become a major challenge in the treatment of nosocomial infections [20,21,22,23], and the impact of antibiotic resistance in ICUs remains extremely high. This is mainly because of the increased usage of different antibiotic classes in ICUs settings [24]. Globally, antibiotic resistance is becoming a serious health challenge [25,26,27]. The world is facing a grave threat of antibiotic resistance, which is increasing with time and present in all countries worldwide, contributing to a huge economic loss [28,29]. The ongoing emergence of antibiotic resistance in the communities and hospitals across India is considered a major threat for public health. Unregulated use of antibiotics is the major reason for increased antibiotic resistance in India [30]. Regularly updated local antibiogram, an essential component of antibiotic stewardship program, can guide the use of empirical antibiotics in critically ill patients and reduce their mortality and morbidity rates [31,32,33].

The important step in reducing the infections is infection control in the ICU, i.e., implementation of antibiotic stewardship, reduction in days of ICU stay, reduction in device days, etc. The mainstay of favorable outcomes in these critically ill patients is timely administration of appropriate empirical antibiotics. However, early use of broad-spectrum antibiotics is a double-edged sword; though it decreases the mortality and morbidity, it is contributing to an increase of antibiotic resistance. This has led to limited therapeutic options to treat multidrug-resistant microorganisms causing infections in the ICU [34].

In our ICU setting in a tertiary care teaching hospital, surveys of nosocomial infections have not been carried out in the recent past. Although the low and middle income countries are hugely burdened with antibiotic resistance, the prevalence and patterns of resistance in ICU patients have not been well documented. The antibiotic resistance patterns vary widely from one country to another, as well as from one hospital to another and even among different ICUs within one hospital. Therefore, the aim of this study was to find out the percentage of different bacterial isolates from the clinical samples of patients admitted in the adult and pediatric ICUs and to obtain the antibiotic resistance patterns in bacterial pathogens so that the right antibiotic choices can be made at the right times.

## 2. Results

A total of 1251 samples were obtained from different patients in the study period. Among these, 480 (38%) of the samples showed bacterial as well as Candida growth. Culture positivity rate among adults and pediatric patients were 37% and 40%, respectively. The most commonly isolated organisms were Gram-negative *Enterobacteriaceae* and non-fermenters than Gram-positive cocci (Table 1).

Maximum culture positivity was found from blood (56%), followed by lower respiratory tract samples (sputum and endotracheal tube aspirates, 27%), and urine (17%). Table 2a shows the total percentage (%) of different microorganisms obtained in adult and pediatric ICUs from different clinical samples such as blood, urine, and respiratory samples.

We found an overall preponderance of culture positivity in males (51%) after analyzing the culture positive data. Interestingly, it was observed that in the adult ICU, samples recovered from female patients showed more culture positivity (52%) than males (48%). However, this was reversed in the pediatric ICU, where males showed preponderance (55%) over females (45%). The same has been depicted in Table 2b.

**Figure 1:**
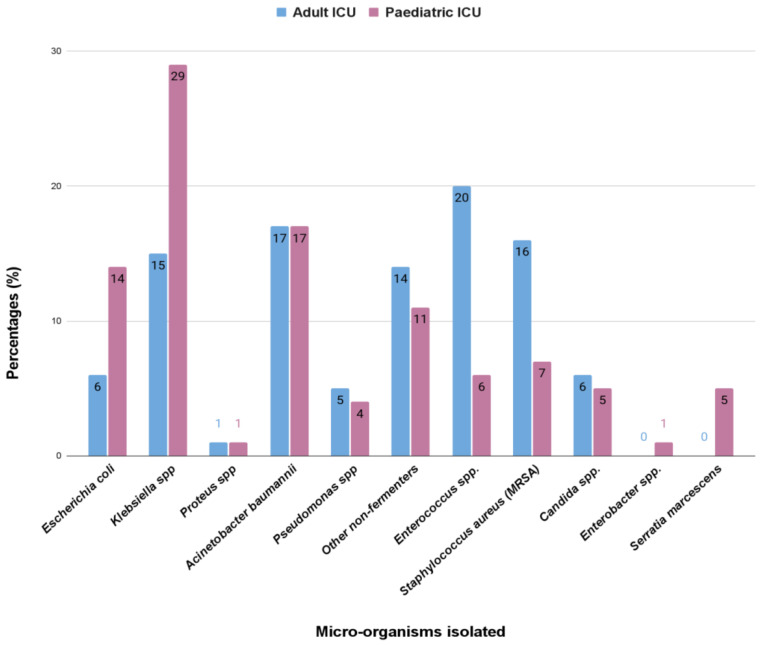
The comparison (in percentage, %) in the pattern of the microorganisms isolated from the bloodstream infections in the adult and pediatric intensive care unit.

We found that in adults, the most common isolated bacteria causing bacteremia was *Enterococcus* spp. (20%), followed by *Acinetobacter* spp., *Staphylococcus aureus*, *Klebsiella pneumoniae*, and other non-fermenters with a prevalence of 17%, 16%, 15%, and 14%, respectively. However, in the pediatric ICUs, the most common cause of bacteremia was by *Klebsiella* spp. (29%), followed by *Acinetobacter, Escherichia coli*, other non-fermenters, and *Staphylococcus aureus*, showing prevalence of 17%, 14%, 11%, and 7%, respectively. The comparison in the pattern of the organisms isolated has been highlighted in the bar diagram in Figure 1.

**Figure 2:**
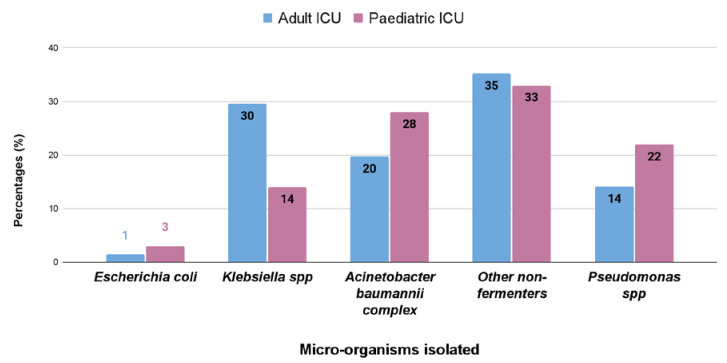
Percentage (%) of different bacterial isolates obtained from respiratory samples of the adult and pediatric intensive care units.

In both adult and pediatricpatients, respiratory tract infection causing pathogens are non-fermentative Gram-negative bacteria, having a prevalence of 35% and 33%, respectively, followed by *Klebsiella* spp. (30%), *Acinetobacter* spp. (20%), and *Pseudomonas* spp. (14%) in adults, and *Acinetobacter* spp. (28%), *Pseudomonas* spp. (22%), and *Klebsiella* spp. (14%) in pediatric patients (Figure 2).

**Figure 3:**
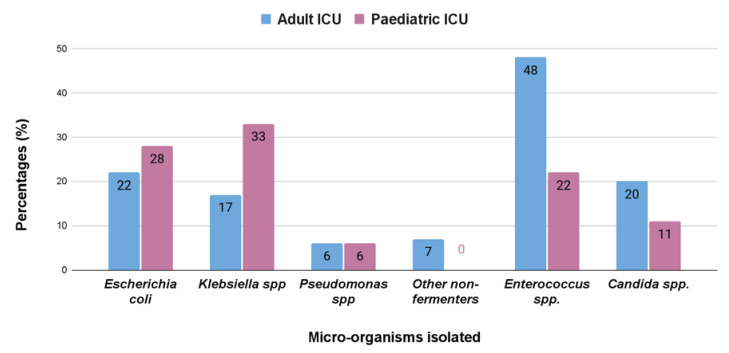
Percentage (%) of different bacterial isolates obtained from the samples from urinary tract infections from adult and pediatric intensive care units.

We found that the isolates obtained from urine showed a preponderance for Gram-positive bacteria in case of adult intensive care unit in contrast to the pediatric intensive care unit where Gram-negative bacteria were more common. *Klebsiella* spp. (33%) were the most common isolate from urinary tract infection in pediatric patients, but in adults it was *Enterococcus* spp. (48%), followed by *Escherichia coli* (22%) and *Klebsiella* spp. (17%) (Figure 3).

### Antibiotic Resistance Patterns

**Figure 4:**
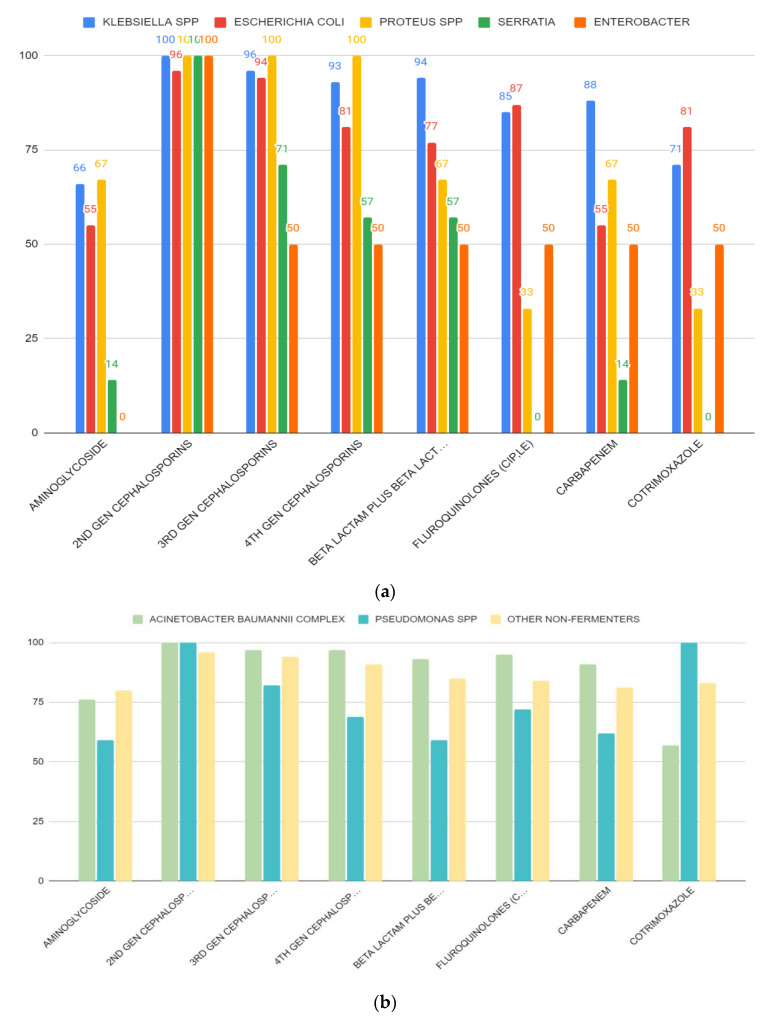
(**a**) Resistance patterns of isolated Enterobacteriaceae from different ICUs against different classes of antibiotics. Most of the microorganisms showed high resistance against cephalosporins. (**b**) Resistance patterns of non-fermenters isolated from different ICUs against some clinically relevant antibiotic classes.

We found that Gram-negative bacterial isolates showed high percentage (%) of antibiotic resistance against different classes of antibiotics. Table 3 and Figure 4 show thepercentage (%) of antibiotic resistance among different Gram-negative bacterial isolates against some clinically relevant antibiotic classes. In this study, *Escherichia coli* from adult patients were found to be resistant to aminoglycosides (75%), second generation cephalosporins (95%), beta-lactams (95%), fluoroquinolones (95%), carbapenems (75%), and cotrimoxazole (85%). *Escherichia coli* from pediatric ICU were found to be resistant to second generation cephalosporins (96%), beta-lactams (63%), fluoroquinolones (81%), and cotrimoxazole (78%). *Klebsiella* spp. from adult patientswere found to be resistant to aminoglycosides (75%), second generation cephalosporins (100%), beta-lactams (94%), fluoroquinolones (92%), carbapenems (88%), and cotrimoxazole (83%). *Proteus* spp. from adult patients were found to be resistant only to cephalosporins (100%). On the other hand, *Proteus* spp. obtained from pediatric patients were found to be resistant to aminoglycosides (100%), second generation cephalosporins (100%), beta-lactams (100%), and carbapenems (100%). *Enterobacter* spp. and *Serratia* spp. obtained from pediatric ICUs showed full resistance against second generation cephalosporins (100%) only. *Acinetobacter baumannii* obtained from both adult and pediatric patients showed high resistance to all antibiotic classes used in this study (Table 3, Figure 4). *Pseudomonas* spp. from adult patients found to be resistant to third generation (100%) and fourth generation cephalosporins (79%), beta-lactams (74%), fluoroquinolones (74%), and carbapenems (89%). Other non-fermenters obtained from both adult and pediatric patients showed high resistance to all antibiotic classes used in this study.

While analyzing the data of antibiotic resistance patterns, we found that *Enterococcus* spp. obtained from adult and pediatric ICUs showed 96% and 92% of resistance against ampicillin, and 98% and 77% of resistance against fluoroquinolone compounds, respectively. MRSA strains from adults showed 55% of resistance against cotrimoxazole. In case of fluoroquinolones, MRSA strains from adult and pediatric patients showed 65% and 60% of resistance, respectively (Table 4).

## 3. Discussion

Nosocomial infections by multidrug-resistant organisms are a worldwide threat as they impose life-threatening risks in critically ill patients and lead to a rise in healthcare cost. Prevention of this ICU infection is the need of the hour. To achieve this, we need to have the knowledge of the local infection rates and the local bacteriological profile along with its antibiotic resistance pattern.

This present study provides data on antibiotic resistance in adult and pediatric ICUs of a tertiary care hospital in Kolkata, India. In this study, we have analyzed a spectrum of pathogens and antibiotic treatments, which allowed a relatively comprehensive documentation of antibiotic resistance in ICU patients.

A total of 1251 samples were obtained from different patients, of which 38% of the samples showed bacterial growth, and 37% and 40% of culture positivity were found from adults and pediatric patients, respectively. Enterococci, one of the members of the ESKAPE group of pathogens, was the most frequent isolate in adults. Gram-negative bacterial infection was predominant in pediatric patients among which non-fermentative Gram-negative rods were most common. Previous studies reported that more than 50% of the nosocomial infections occurring in the hospital ICU were due to Gram-negative bacteria [35,36], which is in accordance with our findings. High occurrence of Gram-negative bacteria in the hospital environment could be the main reason for frequent ICU infections all over the world [34]. Wet environment and waste materials discarded in the sinks or drains of the hospital trigger biofilm formation and these biofilms harbor MDR bacteria, causing nosocomial infections [37]. The occurrence of nosocomial infections changes according to the hospital settings and the surveillance methods used to identify a nosocomial infection [38]. A large multicentric study has reported that at least one ICU acquired infection in 18.9% of patients, with a frequency ranging from 2.3% to 49.2% across the centers [39]. Previously, other studies [40,41] have reported prevalence rates between 9% and 37%, largely based on the patient population studied. In an international study by Vincent et al. [36], involving 1265 ICUs from 76 countries, 51% patients were found with nosocomial infections. However, the rates of such infections varied significantly with the different countries [36]. The patients from a single hospital can have multiple risk of obtaining infections including the severity of illness [35].

Bloodstream infections were the most common infection found in this study (Table 1). In adults, the most common isolated bacteria causing bacteremia was *Enterococcus* spp. (20%), followed by *Acinetobacter* spp. (17%), *Staphylococcus aureus* (16%), *Klebsiella pneumonia* (15%), and other non-fermenters (14%). However, in the pediatric patients, the most common cause of bacteremia was by *Klebsiella* spp. (29%), followed by *Acinetobacter* (17%)*, Escherichia coli* (14%), other non-fermenters (11%), and *Staphylococcus aureus* (7%) (Figure 1). A recent report by Negm et al. [42] demonstrated that bacteremia was the most prevalent in their ICU (32%) patients in Zagazig University Hospitals, Egypt. Respiratory infection (both in ventilated and nonventilated patients) was the second most common infection (27.5%) in both our adult and pediatric populations (Table 2). In both the ICUs, pathogens causing respiratory tract infections are non-fermentative Gram-negative bacteria, having a prevalence of 35% and 33%, respectively (Figure 2). Previously, Dasgupta et al. [12] found that pneumonia was the most frequently detected infection (62.07%) in intensive care unit. According to Shao et al. [43], respiratory tract infections accounted for 64.75% of the total nosocomial infections in the First Affiliated Hospital in Zhejiang Province, China. We also found, in case of urinary tract infection, that *Klebsiella* spp. (33%) were the most common isolate from urinary tract infection in pediatric patients, but in adults it was *Enterococcus* spp. (48%), followed by *Escherichia coli* (22%), and *Klebsiella* spp. (17%) (Figure 3). According to the United States National Nosocomial Infections Surveillance System, the frequency of different nosocomial infections can vary with different studies in different hospital settings [35].

An important finding from this current study was the extent of antibiotic resistance among key pathogens. Interestingly, most of the bacterial isolates showed very high resistance against different antibiotic classes (Table 3 and Table 4, Figure 4a,b). This is of serious public health concern as these antibiotic classes represent the most important antibiotics to treat nosocomial infections. Antibiotic-resistant ESKAPE (*Enterococcus faecium*, *Staphylococcus aureus*, *Klebsiella pneumoniae*, *Acinetobacter baumannii*, *Pseudomonas aeruginosa*, and *Enterobacter* species) pathogens represent a global threat to human health and the health care system. The acquisition of antibiotic resistance genes by ESKAPE pathogens has gradually reduced the treatment options for serious nosocomial infections, increased the burden of infectious diseases, and increased mortality rates due to the treatment failure in hospitals [15]. Recently, Tran GM et al. [44] described the patterns of antibiotic resistance in ICU patients admitted in a tertiary hospital in Vietnam. Their study was focused on Ventilator-associated pneumonia (VAP). *Pseudomonas aeruginosa* and *Acinetobacter baumannii* resulted to be resistant to ceftazidime, ceftriaxone, piperacillin, imipenem, meropenem, ertapenem, ciprofloxacin and levofloxacin; moreover, high rates (>70%) of ceftriaxone and ceftazidime-resistant *Klebsiella* spp. were also observed [44]. The emergence of multidrug-resistant non-fermentative Gram-negative bacterial infection in hospitalized patients in a tertiary care center of Nepal was also reported recently by Yadav et al. [45]. The MDR bacteria can be easily transmitted to different patient groups admitted in the ICUs either from contaminated places or from contaminated hands or equipment used in the hospital. Cross-infections due to transmission of bacteria can contribute to a rise in nosocomial infections during a long hospital stay. Rise in MDR bacterial strains in the ICUs in hospital settings could be the result of inappropriate antibiotic uses, a lack of proper maintenance of hygiene, inappropriate cleaning of the hospital environment, and, most importantly, newer methods of antibiotic-resistant mechanisms acquired by the opportunistic pathogenic bacteria [46]. Therefore, the establishment of the local antibiogram, regular surveillance of the nosocomial infection rates, and extensive cleaning of the ICU environment are mainstays of combating ICU infections by multidrug-resistant organisms.

For empirical therapy, we suggested the use of combination therapy including one bactericidal and one bacteriostatic drug as per the sensitivity pattern of the hospital antibiogram. There is further scope of study on fractional inhibitory concentration to determine the synergism between the two antibiotics.

## 4. Materials and Methods

### 4.1. Sample Collection

A cross-sectional, retrospective study was conducted from January2022 to October 2022 on a total of 139 adult and 146 pediatric patients who were admitted to the ICUs of a tertiary care hospital, Medical College and Hospital (MCH) in Kolkata, India. Detailed history and associated risk factors of these patients were taken. The total number of clinical specimens received were 695 from adult ICU and 556 from pediatric ICU. An intensive care unit is one of the hospital wards which is critical in the treatment of many serious diseases. All the clinical samples were collected appropriately, following the standard protocol [47] at the bedside, and were then transported to the Microbiology Laboratory of the Department of Microbiology, Medical College and Hospital, Kolkata, India, as early as possible. The clinical specimens that were processed include blood, urine, sputum, endotracheal tube (ET) aspirate, and swabs from central line catheter insertion sites. For the purposes of Centers for Disease Control and Prevention (CDC)/National Healthcare Safety Network (NHSN) surveillance in the acute care setting, a healthcare-associated infection is a localized or systemic condition resulting from an adverse reaction to the presence of an infectious agent(s) or its toxin(s) that was not present on admission to the acute care facility. Traditionally, a time cut-off of 48 h after admission is used to differentiate between hospital and community acquired infections [48].

### 4.2. Bacterial Culture

Blood cultures were inoculated in BacT/ALERT bottle for aerobic culture. Positive flagged blood culture bottles and other samples were initially inoculated on blood agar and MacConkey agar and incubated aerobically at 37 °C overnight. The bacterial species were identified by the colony characteristics on MacConkey and blood agar, Gram staining, motility, routine rapid tests such as catalase and oxidase, and routine biochemical tests such as indole, triple sugar iron agar, urease test, and citrate utilization test.

### 4.3. Antibiotic Susceptibility Test (AST)

Antibiotic susceptibility was performed on Mueller Hinton agar following Clinical and Laboratory Standards Institute (CLSI) guidelines [49]. A total of seven classes of antibiotics were tested for all microorganisms that included the following: (a) penicillins, such as ampicillin (10µg); (b) beta-lactam-beta-lactamase inhibitor combination, such as piperacillin/tazobactam (100/10 µg), and amoxycillin/clavulanic Acid (20/10 µg); (c) cephalosporins, such as cefepime (30 µg), cefotaxime (30 µg), cefuroxime (30 µg), ceftriaxone (30 µg), and ceftazidime (30 µg); (d) aminoglycosides, such as amikacin (30 µg) and gentamicin (10 µg); (e) carbapenems, such as imipenem (10 µg) and meropenem (10 µg); (f) fluoroquinolones, such as ciprofloxacin (5 µg) and levofloxacin (5 µg); and (g) trimethoprim/sulfamethoxazole (1.25/23.75 µg). Further, identification of the organism and their susceptibility and resistance patterns were assessed by the automated system VITEK 2 compact system (bioMerieux), according to manufacturer’s instruction. Results were interpreted according to the Clinical and Laboratory Standards Institute (CLSI) criteria [49]. Methicillin-resistant Staphylococcus aureus was detected by both antibiotic disc diffusion assay and by VITEK 2. Vancomycin-resistant Enterococcus was also detected by both disc diffusion assay and VITEK 2. For MRSA diagnosis, cefoxitin (disc content 30 µg) screening test was done on Muller Hinton agar with 0.5 McFarland standard inoculum and zone diameter of ≤21 mm were taken as MRSA as per CLSI guidelines. Results were then corroborated with cefoxitin screening test of VITEK 2 compact system. Similarly, for determination of vancomycin resistant Enterococcus, disc diffusion method (disc content 30 µg) was done on Muller Hinton agar and zone diameter of ≤14 mm was taken as VRE as per CLSI guidelines [49]. Results were again corroborated with those of VITEK 2 compact system. VITEK cards used were VITEK 2 GP ID & AST-P628 and VITEK 2 GN ID & AST-N280.

### 4.4. Data Analysis

All patients’ data were electronically stored in a relational database system specifically developed for the study. The data were then exported into a spreadsheet for further analysis. The prevalence of antibiotic resistance was estimated as the proportion of positive results over the entire study sample. Multi-drug resistance (MDR) was defined as resistant to at least three antibiotic classes.

## 5. Conclusions

Our study demonstrated the burden of antibiotic resistance in adult and pediatric ICUs in a tertiary care hospital. Gram-negative Enterobacteriaceae and non-fermenters were found to be the most commonly isolated pathogens in adult and pediatric ICUs than Gram-positive cocci. Bacterial pathogens obtained from ICUs showed high resistance against different clinically relevant antibiotic classes. The high rate of multidrug resistance observed is a deterrent to the management of critically ill patients. Our findings have significant clinical implications for the treatment and management of ICU patients. Minimizing the use of broad-spectrum antibiotics and the appropriate usage of antibiotics during the treatment of patients with nosocomial infections would help to reduce the problem with antibiotic resistance in hospital settings. We suggest the use of combination therapy consisting of one bactericidal and one bacteriostatic drug as per the hospital antibiogram for empirical therapy. Establishing an active surveillance system along with a proper infection control programme will be helpful in combating antibiotic resistance incidence in hospital ICUs.

## Figures and Tables

**Table 1:** The percentage (%) of different groups of microorganisms obtained from adult and pediatric ICUs.

	Adult ICUs		Pediatric ICUs	
Microorganisms	Numbers Obtained (Total = 258)	Percentage (%)	Numbers Obtained (Total = 222)	Percentage (%)
Enterobacteriaceae	69	27	94	42
Non-fermenters	100	39	96	43
Gram-positive cocci	71	28	23	10
Candida	18	6	9	4

**Table 2:** (**a**) The percentage (%) of different microorganisms obtained from adult and pediatric ICU from blood, urine, and respiratory samples. (**b**) The percentage culture positivity (%) in male and female patients in adult and pediatric ICU.

a					
	Blood		Urine	Respiratory Sample	
Adult ICU	122		65	71	
Pediatric ICU	146		18	58	
Total (n)	268		83	129	
Percentage (%)	56		17	27	
**b**					
**Type of ICU**	**Total Number of Positive Cultures (n)**	**Number of Positive Samples in Male**	**Percentage Positivity in Male (%)**	**Number of Positive Samples in Female**	**Percentage Positivity in Female (%)**
Adult ICU	258	124	48 (124/258)	134	52 (134/258)
Pediatric ICU	222	121	55 (121/222)	101	45 (101/222)

**Table 3:** Percentage (%) of antibiotic resistance among different Gram-negative bacterial isolates from both adult and pediatric ICUs.

Microorganisms	Antibiotic Classes															
	Aminoglycosides		2nd Generation Cephalosporins		3rd Generation Cephalosporins		4th Generation Cephalosporins		Beta Lactam-Beta Lactam Inhibitors		Fluoroquinolones		Carbapenems		Cotrimoxazole	
	Adult ICU	Pediatric ICU	Adult ICU	Pediatric ICU	Adult ICU	Pediatric ICU	Adult ICU	Pediatric ICU	Adult ICU	Pediatric ICU	Adult ICU	Pediatric ICU	Adult ICU	Pediatric ICU	Adult ICU	Pediatric ICU
*Escherichia coli*	75 (15/20)	41 (11/27)	95 (19/20)	96 (26/27)	95 (19/20)	93 (25/27)	95 (19/20)	70 (19/27)	95 (19/20)	63 (17/27)	95 (19/20)	81 (22/27)	75 (15/20)	41 (11/27)	85 (17/20)	78 (21/27)
*Klebsiella* spp.	75 (36/48)	59 (33/56)	100 (48/48)	100 (56/56)	96 (46/48)	96 (54/56)	92 (44/48)	95 (53/56)	94 (45/48)	95 (53/56)	92 (44/48)	79 (44/56)	88 (42/48)	88 (49/56)	83 (40/48)	61 (34/56)
*Proteus* spp.	0 (0/1)	100 (2/2)	100 (1/1)	100 (2/2)	100 (1/1)	100 (2/2)	100 (1/1)	100 (2/2)	0 (0/1)	100 (2/2)	0 (0/1)	50 (1/2)	0 (0/1)	100 (2/2)	0 (0/1)	50 (1/2)
*Serratia* spp.	n/a	14 (1/7)	n/a	100 (7/7)	n/a	71 (5/7)	n/a	57 (4/7)	n/a	57 (4/7)	n/a	0 (0/7)	n/a	14 (1/7)	n/a	0 (0/7)
*Enterobacter* spp.	n/a	0 (0/2)	n/a	100 (2/2)	n/a	50 (1/2)	n/a	50 (1/2)	n/a	50 (1/2)	n/a	50 (1/2)	n/a	50 (1/2)	n/a	50 (1/2)
*Acinetobacter baumannii complex*	66 (23/35)	85 (35/41)	100 (35/35)	100 (41/41)	97 (34/35)	98 (40/41)	97 (34/35)	98 (40/41)	91 (32/35)	95 (39/41)	97 (34/35)	93 (38/41)	89 (31/35)	93 (38/41)	86 (30/35)	32 (13/41)
*Pseudomonas* spp.	36 (12/19)	55 (11/20)	n/a	n/a	100 (12/19)	65 (13/20)	79 (15/19)	60 (12/20)	74 (14/19)	45 (9/20)	74 (14/19)	70 (14/20)	89 (17/19)	35 (7/20)	n/a	n/a
*Other non-fermenters*	83 (38/46)	77 (27/35)	100 (46/46)	91 (32/35)	93 (43/46)	94 (33/35)	91 (42/46)	91 (32/35)	91 (42/46)	77 (27/35)	85 (39/46)	83 (29/35)	89 (41/46)	71 (25/35)	87 (40/46)	77 (27/35)

**Table 4:** Percentage (%) of antibiotic resistance among different Gram-positive bacterial isolates obtained from both adult and pediatric ICUs.

Antibiotic Classes														
Microoganisms	Ampicillin		Cotrimoxazole		Vancomycin		Linezolid		Aminoglycosides		High Level Gentamicin		Fluoroquinolones	
	Adult ICU	Pediatric ICU	Adult ICU	Pediatric ICU	Adult ICU	Pediatric ICU	Adult ICU	Pediatric ICU	Adult ICU	Pediatric ICU	Adult ICU	Pediatric ICU	Adult ICU	Pediatric ICU
*Staphylococcus aureus (MRSA)*	n/a	n/a	55 (11/20)	0 (0/10)	0 (0/20)	0 (0/10)	0 (0/20)	0 (0/10)	30 (6/20)	40 (4/10)	n/a	n/a	65 (13/20)	60 (6/10)
*Enterococcus* spp.	96 (49/51)	92 (12/13)	0 (51/51)	0 (13/13)	33 (17/51)	31 (4/13)	8 (4/51)	0 (0/13)	n/a	n/a	43 (22/51)	31 (4/13)	98 (90/95)	77 (10/13)

## Data Availability

Not applicable.

## References

[1] Lambert M.-L., Suetens C., Savey A., Palomar M., Hiesmayr M., Morales I., Agodi A., Frank U., Mertens K., Schumacher M. (2011). Clinical outcomes of health-care-associated infections and antimicrobial resistance in patients admitted to European intensive-care units: A cohort study. Lancet Infect. Dis..

[2] Bereket W., Hemalatha K., Getenet B., Wondwossen T., Solomon A., Zeynudin A., Kannan S. (2012). Update on bacterial nosocomial infections. Eur. Rev. Med. Pharmacol. Sci..

[3] World Health Organization (2002). Prevention of Hospital-Acquired Infection: A Practical Guide (2nd Edition). https://apps.who.int/iris/handle/10665/67350.

[4] Blot S., Depuydt P., Vandewoude K., De Bacquer D. (2007). Measuring the impact of multidrug resistance in nosocomial infection. Curr. Opin. Infect. Dis..

[5] Carlet J., Ben Ali A., Tabah A., Willems V., Philippart F., Chafine A., Garrouste-Orgeas M., Misset B., Kuhlen R., Moreno R., Ranieri V.M., Rhodes A. (2007). Multidrug resistant infections in the ICU: Mechanisms, prevention and treatment. 25 Years of Progress and Innovation in Intensive Care Medicine.

[6] Pittet D., Tarara D., Wenzel R.P. (1994). Nosocomial bloodstream infection in critically ill patients. Excess length of stay, extra costs, and attributable mortality. JAMA.

[7] Anand N., Nayak I.M., Advaitha M.V., Thaikattil N.J., Kantanavar K.A., Anand S. (2016). Antimicrobial agents’ utilization and cost pattern in an intensive care unit of ateaching hospital in South India. Indian J. Crit. Care Med..

[8] Campion M., Scully G. (2018). Antibiotic Use in the Intensive Care Unit: Optimization and De-Escalation. J. Intensive Care Med..

[9] Torres A., Aznar R., Gatell J.M., Jiménez P., González J., Ferrer A., Celis R., Rodriguez-Roisin R. (1990). Incidence, Risk, and Prognosis Factors of Nosocomial Pneumonia in Mechanically Ventilated Patients. Am. Rev. Respir. Dis..

[10] Young L.S. (1981). Nosocomial infections in the immunocompromised adult. Am. J. Med..

[11] Hanson L.C., Weber D.J., Rutala W.A., Samsa G.P. (1992). Risk factors for nosocomial pneumonia in the elderly. Am. J. Med..

[12] Hazra A., Dasgupta S., Das S., Chawan N.S. (2015). Nosocomial infections in the intensive care unit: Incidence, risk factors, outcome and associated pathogens in a public tertiary teaching hospital of Eastern India. Indian J. Crit. Care Med..

[13] Santajit S., Indrawattana N. (2016). Mechanisms of Antimicrobial Resistance in ESKAPE Pathogens. BioMed Res. Int..

[14] Mulani M.S., Kamble E.E., Kumkar S.N., Tawre M.S., Pardesi K.R. (2019). Emerging Strategies to Combat ESKAPE Pathogens in the Era of Antimicrobial Resistance: A Review. Front. Microbiol..

[15] De Oliveira D.M., Forde B.M., Kidd T.J., Harris P.N., Schembri M.A., Beatson S.A., Paterson D.L., Walker M.J. (2020). Antimicrobial Resistance in ESKAPE Pathogens. Clin. Microbiol. Rev..

[16] Rice L.B. (2008). Federal Funding for the Study of Antimicrobial Resistance in Nosocomial Pathogens: No ESKAPE. J. Infect. Dis..

[17] Navidinia M. (2016). The clinical importance of emerging ESKAPE pathogens in nosocomial infections. J. Paramed. Sci..

[18] European Centre for Disease Prevention and Control (2017). Surveillance of Healthcare-Associated Infections and Prevention Indicators in European Intensive Care Units: HAI-Net ICU Protocol, Version 2.2.

[19] Branstetter J.W., Barker L., Yarbrough A., Ross S., Stultz J.S. (2021). Challenges of Antibiotic Stewardship in the Pediatric and Neonatal Intensive Care Units. J. Pediatr. Pharmacol. Ther..

[20] Kollef M.H., Fraser V.J. (2001). Antibiotic resistance in the intensive care unit. Ann. Intern. Med..

[21] Silveira F., Fujitani S., Paterson D.L. (2004). Antibiotic-resistant infections in the critically ill adult. Clin. Lab. Med..

[22] Brusselaers N., Vogelaers D., Blot S. (2011). The rising problem of antimicrobial resistance in the intensive care unit. Ann. Intensive Care.

[23] Pons M.J., Ruiz J. (2019). Current trends in epidemiology and antimicrobial resistance in intensive care units. J. Emerg. Crit. Care Med..

[24] Krivoy N., El-Ahal W.A., Bar-Lavie Y., Haddad S. (2007). Antibiotic prescription and costpatterns in a general intensive care unit. Pharm. Pract..

[25] O’Neill J. (2014). Review on Antimicrobial Resistance: Tackling a Crisis for the Health and Wealth of Nations.

[26] Centres for Disease Control and Prevention (CDC) (2019). Antibiotic Resistance Threats in the United States.

[27] World Health Organization (2015). Global Action Plan on Antimicrobial Resistance.

[28] French G.L. (2005). Clinical impact and relevance of antibiotic resistance. Adv. Drug Deliv. Rev..

[29] Michael C.A., Dominey-Howes D., Labbate M. (2014). The Antimicrobial Resistance Crisis: Causes, Consequences, and Management. Front. Public Health.

[30] Savanur S.S., Gururaj H. (2019). Study of Antibiotic Sensitivity and Resistance Pattern of Bacterial Isolates in Intensive Care Unit Setup of a Tertiary Care Hospital. Indian J. Crit. Care Med..

[31] Vincent J.-L., Bassetti M., François B., Karam G., Chastre J., Torres A., Roberts J.A., Taccone F.S., Rello J., Calandra T. (2016). Advances in antibiotic therapy in the critically ill. Crit. Care.

[32] Hilnani G.C., Zirpe K., Hadda V., Mehta Y., Madan K., Kulkarni A., Mohan A., Dixit S., Guleria R., Bhattacharya P. (2019). Guidelines for Antibiotic Prescription in Intensive Care Unit. Indian J. Crit. Care Med..

[33] Luyt C.E., Bréchot N., Trouillet J.L., Chastre J. (2014). Antibiotic stewardship in the intensive care unit. Critical Care.

[34] Qadeer A., Akhtar A., Ain Q.U., Saadat S., Mansoor S., Assad S., Ishtiaq W., Ilyas A., Khan A.Y., Ajam Y. (2016). Antibiogram of Medical Intensive Care Unit at Tertiary Care Hospital Setting of Pakistan. Cureus.

[35] Richards M.J., Edwards J.R., Culver D.H., Gaynes R.P., National Nosocomial Infections Surveillance System (2000). Nosocomial Infections in Combined Medical-Surgical Intensive Care Units in the United States. Infect. Control. Hosp. Epidemiol..

[36] Vincent J.L., Rello J., Marshall J., Siva E., Anzueto A., Martin C.D., Moreno R., Lipman J., Sakr Y., Reinhart K. (2009). The extended prevalence of infection in the ICU study: EPIC II. JAMA.

[37] Chia P.Y., Sengupta S., Kukreja A., Ponnampalavanar S.S., Ng O.T., Marimuthu K. (2020). The role of hospital environment in transmissions of multidrug-resistant gram-negative organisms. Antimicrob. Resist. Infect. Control.

[38] Gastmeier P., Sohr D., Just H.-M., Nassauer A., Daschner F., Ruden H. (2000). How to Survey Nosocomial Infections. Infect. Control. Hosp. Epidemiol..

[39] Alberti C., Brun-Buisson C., Burchardi H., Martin C., Goodman S., Artigas A., Sicignano A., Palazzo M., Moreno R., Boulmé R. (2002). Epidemiology of sepsis and infection in ICU patients from an international multicentre cohort study. Intensive Care Med..

[40] Rebollo M.H., Bernal J.M., Llorca J., Rabasa J.M., Revuelta J.M. (1996). Nosocomial infections in patients having cardiovascular operations: A multivariate analysis of risk factors. J. Thorac. Cardiovasc. Surg..

[41] Papia G., McLellan B.A., El-Helou P., Louie M., Rachlis A., Szalai J.-P., Simor A.E. (1999). Infection in Hospitalized Trauma Patients: Incidence, Risk Factors, and Complications. J. Trauma Acute Care Surg..

[42] Negm E.M., Mowafy S.M.S., Mohammed A.A., Amer M.G., Tawfik A.E., Ibrahim A.E.S., Hassan T.H. (2021). Antibiograms of intensive care units at an Egyptian tertiary care hospital. Egypt. J. Bronchol..

[43] Shao L.-W., Ni L.-M., Gao C.-H., Wei J.-H., Zhong Z.-F., Meng S.-Q., Yang W.-B., Liu J.H. (2016). The incidence and risk factors of nosocomial infections in intensive care unit in China: An epidemiological study of 1718 patients. Int. J. Clin. Exp. Med..

[44] Tran G.M., Ho-Le T.P., Ha D.T., Tran-Nguyen C.H., Nguyen T.S.M., Pham T.T.N., Nguyen D.A., Hoang H.Q., Tran N.V., Nguyen T.V. (2017). Patterns of antimicrobial resistance in intensive care unit patients: A study in Vietnam. BMC Infect. Dis..

[45] Yadav S.K., Bhujel R., Mishra S.K., Sharma S., Sherchand J.B. (2020). Emergence of multidrug-resistant non-fermentative gram negative bacterial infection in hospitalized patients in a tertiary care center of Nepal. BMC Res. Notes.

[46] Uc-Cachón A.H., Gracida-Osorno C., Luna-Chi I.G., Jiménez-Guillermo J.G., Molina-Salinas G.M. (2019). High Prevalence of Antimicrobial Resistance among Gram-Negative Isolated Bacilli in Intensive Care Units at a Tertiary-Care Hospital in Yucatán Mexico. Medicina.

[47] Patricia M.T. (2021). Bailey & Scott’s Diagnostic Microbiology.

[48] Kouchak F., Askarian M. (2012). Nosocomial Infections: The Definition Criteria. Iran. J. Med. Sci..

[49] CLSI (2020). Performance Standards for Antimicrobial Susceptibility Testing.

